# Proactive interventions for smoking cessation in general medical practice: a quasi-randomized controlled trial to examine the efficacy of computer-tailored letters and physician-delivered brief advice*

**DOI:** 10.1111/j.1360-0443.2007.02031.x

**Published:** 2008-02

**Authors:** Christian Meyer, Sabina Ulbricht, Sebastian E Baumeister, Anja Schumann, Jeannette Rüge, Gallus Bischof, Hans-Jürgen Rumpf, Ulrich John

**Affiliations:** 1 University of Greifswald, Department of Epidemiology and Social Medicine Greifswald, Germany; 2 Bremen Institute for Prevention Research and Social Medicine Bremen, Germany; 3 University of Lübeck, Department of Psychiatry and Psychotherapy, Research Group S:TEP (Substance Abuse: Treatment, Epidemiology and Prevention) Lübeck, Germany

**Keywords:** Computer expert system, opportunistic counselling, primary medical care, smoking cessation, Transtheoretical Model

## Abstract

**Aims:**

To test the efficacy of (i) computer-generated tailored letters and (ii) practitioner-delivered brief advice for smoking cessation against an assessment-only condition; and to compare both interventions directly.

**Design:**

Quasi-randomized controlled trial.

**Setting:**

A total of 34 randomly selected general practices from a German region (participation rate 87%).

**Participants:**

A total of 1499 consecutive patients aged 18–70 years with daily cigarette smoking (participation rate 80%).

**Interventions:**

The tailored letters intervention group received up to three individualized personal letters. Brief advice was delivered during routine consultation by the practitioner after an onsite training session. Both interventions were based on the Transtheoretical Model of behaviour change.

**Measurements:**

Self-reported point prevalence and prolonged abstinence at 6-, 12-, 18- and 24-month follow-ups.

**Findings:**

Among participants completing the last follow-up, 6-month prolonged abstinence was 18.3% in the tailored letters intervention group, 14.8% in the brief advice intervention group and 10.5% in the assessment-only control group. Assuming those lost to follow-up to be smokers, the rates were 10.2%, 9.7% and 6.7%, respectively. Analyses including all follow-ups confirmed statistically significant effects of both interventions compared to assessment only. Using complete case analysis, the tailored letters intervention was significantly more effective than brief advice for 24-hour [odds ratio (OR) = 1.4; *P* = 0.047] but not for 7-day point prevalence abstinence (OR = 1.4; *P* = 0.068) for prolonged abstinence, or for alternative assumptions about participants lost to follow-up.

**Conclusions:**

The study demonstrated long-term efficacy of low-cost interventions for smoking cessation in general practice. The interventions are suitable to reach entire populations of general practices and smoking patients. Computer-generated letters are a promising option to overcome barriers to provide smoking cessation counselling routinely.

## INTRODUCTION

Assisting patients to quit smoking is regarded as one of the most cost-effective measures in clinical practice [1]. A recent review confirmed that simple interventions, such as brief advice delivered by a physician, have a small but significant effect on cessation rates [2]. From a public health perspective, implementing interventions in primary medical care is promising, because the majority of the smoking population can be reached within 1 year. Therefore, even interventions with a small effect are capable of substantially reducing smoking-attributable disease at population level [3]. Systematic screening and advising patients on smoking cessation on a routine basis has been adopted by clinical practice guidelines [4,5].

However, these recommendations are implemented insufficiently in practice [6–11]. Lack of time and patients' insufficient motivation to change have been reported as major barriers against the provision of smoking interventions by practitioners [11–14]. The provision of time-saving opportunistic counselling approaches allowing the inclusion of all patients irrespective of readiness to change smoking behaviour can be expected to be crucial for improving implementation [15].

Computer-based smoking interventions are alternatives to interpersonal counselling [16]. Such interventions comprised one or more letters tailored to characteristics of the individual smoker [17]. There is evidence that tailored self-help materials are more effective than no intervention or untailored materials [18]. However, studies testing tailored self-help materials in primary medical care settings revealed mixed results [19–22]. So far, no efficacy trial has been provided that allowed direct comparison of physician-delivered counselling and computer-tailored self-help materials in the general practice setting. The objective of the present study is to test the short- and long-term efficacy of brief advice, which is delivered by a representative sample of general practitioners, and computer-generated tailored letters in a randomly selected population of adult smoking patients.

## METHOD

As part of the project ‘Proactive interventions for smoking cessation in General medical Practices’ (Pro GP) we conducted a randomized controlled trial comparing the effect of a brief advice delivered by practitioners who were trained in opportunistic counselling techniques, and up to three computer-generated tailored letters, each against assessment only.

### Sampling

Two-step sampling was used to generate a representative sample of out-patients. In Germany, primary medical care is provided by general practitioners (GPs) who work in private practices and are paid by fee for service from health insurance companies. Almost all the population has public or private health insurance.

In a first step, 44 of 149 practices were selected randomly from all practitioners registered for primary medical care in Vorpommern, a rural area in north-east Germany. Five practices did not provide primary care and 34 of the remaining 39 (87.2%) took part. They included 39 GPs.

In a second step, for a period of 3 weeks all consecutive patients were screened for smoking status by a research nurse covering complete office hours. In total, we registered 11 560 practice attendances (Table 1). The number of consultations varied across study weeks because of seasonal effects. Patients visiting the practice repeatedly within the study period (*n* = 1664) were subsequently excluded from re-recruitment, leaving 9896 patients. We excluded 7696 patients who did not fulfil the inclusion criteria, i.e. age below 18 or above 70 years or not smoking daily in the past 4 weeks. Further, 383 patients were excluded for several reasons (e.g. too ill, cognitively impaired, insufficient language capabilities, screening refused or missed). Among patients fulfilling the inclusion criteria (*n* = 1862), 1499 (80.5%) consented to take part. Recruitment took place from April 2002 to September 2003.

**Table 1:** Recruitment and retention of participants.

	First study week ‘assessment only’ n (%)	Second study week ‘tailored letters’ n (%)	Third study week ‘brief advice’ n (%)
Consultations*	4290	3836	3434
Patients (% of consultations)†	3993 (93.1)	3229 (84.2)	2674 (77.9)
Fulfilled inclusion criteria (% of patients)	756 (18.9)	602 (18.6)	504 (18.8)
Agreed to participate (% of patients fulfilling inclusion criteria)	609 (80.6)	488 (81.1)	402 (79.8)
Participated in follow-up (% of patients agreeing to participate):			
Month 3	–	389 (79.7)	–
Month 6	480 (78.8)	333 (68.2)	324 (80.6)
Month 12	453 (74.4)	319 (65.4)	311 (77.4)
Month 18	419 (68.8)	302 (61.9)	283 (70.4)
Month 24	396 (65.0)	279 (57.2)	266 (66.2)

A flow diagram detailing reasons for exclusion and non-participation is available on request.

* Consultations included multiple practice visits of the same patients.

† Patients recurrently visiting the practice were allocated to the group belonging to the time of the first practice visits.

### Assignment

We utilized quasi-randomization based on the time of practice attendance: (i) patients in the first study week were allocated to the assessment-only control condition; (ii) patients in the second week were assigned to the tailored letters intervention; and (iii) patients in the third week were assigned to the brief advice intervention. We chose a fixed sequence of study conditions to avoid counselling activities of the practitioner in the first and second weeks. The practitioner received counselling training between study weeks 2 and 3. To reduce potential bias associated with patients attending the practice repeatedly, the study weeks were scheduled at least 2 weeks apart. However, patients attending the practice frequently still had a lower probability of inclusion in the later study groups (Table 1).

### Interventions

#### Tailored letters

The letters were tailored according to the principles of the Transtheoretical Model (TTM) of behaviour change [23]. The letters were generated at our research institute by a computer expert system, which was structurally comparable to the system developed by Velicer *et al*. [16]. The system used in this study was based on an initial version provided by the Cancer League Switzerland [24], which was reprogrammed and modified with respect to feedback paragraphs and norm data. Letters were tailored to the stage of change, defined by current smoking status and intention to quit smoking and scores on decisional balance, self-efficacy and processes of change questionnaires. The first letter, which was based on data gathered at the baseline assessment, included normative feedback, i.e. feedback that depends on the individual scores compared to the population norm by stage, and was sent out within 1 week after the practice visit. The 3-month and 6-month letters additionally included ipsative feedback, i.e. information that is tailored to individual change since the previous assessment of the different constructs. The letters were accompanied by a selection from a series of self-help manuals covering specific information relevant for the particular stage of change according to the TTM. No information about the participants was given to the practice team or the practitioner.

#### Brief advice

A 2-hour onsite training session was provided for each practitioner by a researcher. The first part comprised an introduction to the epidemiology of smoking and smoking-related disease, nicotine dependence, an overview of evidence-based interventions for smoking cessation and principals of the TTM. In the second part we introduced the brief advice intervention and the study protocol for the following study week. We adopted elements of health behaviour change counselling [25]. The intervention was structured by a desktop resource providing a flow chart illustrating adequate elements for the counselling session and general communication strategies. To prompt the counselling, basic information about smoking-related variables (e.g. cigarettes smoked per day, degree of nicotine dependence, carbon monoxide in exhaled air, stage of change) from the assessment in the waiting room was given to the practitioner on a summary-sheet, matched to the desktop resource. The intervention was designed to last 10 minutes and included the same self-help manuals as those provided for patients in the tailored letters condition. The intervention was delivered by the practitioner at the same visit within the regular consultation following the baseline assessment in the waiting room.

#### Assessment only

No intervention beside usual practice routine was provided for the control group. No information about the participants was given to the practice team or the practitioner and no self-help manuals have been provided.

### Assessment

For the baseline assessment a 22-sided questionnaire was administered in the waiting room, including items covering socio-economic status, smoking behaviour, TTM and other psychological constructs regarding smoking, psychosocial resources and a screening for alcohol use disorders. For the present analysis general health was assessed by the EuroQol visual analogue scale (EQ-VAS) [26]. The Fagerstöm Test for Nicotine Dependence (FTND) was used to determine degree of nicotine dependence [27]. Carbon monoxide concentration in exhaled air was measured by the research nurse [28].

We conducted outcome assessments 6, 12, 18 and 24 months after the initial practice visit. An additional assessment was performed after 3 months in the tailored letters condition for the purpose of the intervention. The follow-up assessments included items covering the TTM constructs, smoking behaviour, utilization of medical care and smoking cessation aids and quality of life. According to recent consensus we considered the following self-reported point prevalence abstinence and prolonged abstinence measures as the primary outcome measures [29]: (i) 24-hour point prevalence abstinence (i.e. not smoking a puff within the past 24 hours preceding the follow-up); (ii) 7-day point prevalence abstinence (i.e. not smoking a puff within the past 7 days preceding the follow-up); (iii) 4-week prolonged abstinence (i.e. not smoking in the past 4 weeks preceding the follow-up); (iv) 6-month prolonged abstinence (i.e. not smoking in the past 6 months preceding the follow-up). Outcome assessment was collected via computer-assisted telephone interview if possible. As part of a standard procedure established at the survey unit of our institution, several measures to ensure high-quality data have been used. This included double entry of paper-and-pencil questionnaires, regular training, monitoring and supervision of interviewers by experienced research psychologists and automated consistence checks during interviewing. The interviewers were blind to treatment allocation and not involved in the handling of the tailored letter. A questionnaire was sent out if a participant could not be reached by telephone. The response rates are shown in Table 1, with no contact being the main reason for non-response. Refusal to participate ranged from 8.8% (6 months) to 3.7% (24 months) of the participants lost at follow-up.

### Statistical analysis

We analysed differences with respect to potential confounders at baseline and smoking cessation at follow-up between both intervention groups and the assessment-only group. To take clustering within practices into account, we applied the sample survey methods in STATA version 9.2 [30]. Associations between smoking abstinence and treatment groups were estimated using a logistic generalized estimating equation (GEE). The PROC GENMOD procedure in SAS version 9.1 was used because it allows specifying two levels of clustering (practice site and individual). To examine the implications of missing data and sample attrition for study conclusions, a series of additional analyses were undertaken. First, regression imputation methods were used to impute missing data on covariates measured at baseline, and all covariate adjustment models were computed with the missing data replaced by the imputed values. The regression imputation was conducted using the ICE procedure of STATA [31]. Secondly, we conducted a series of analyses making four different assumptions about those who dropped out: (i) a complete cases analysis (i.e. only those whose smoking status at a follow-up was known were included in the model); (ii) all missing follow-up observations were coded as smokers; (iii) the last value was carried forward to replace the missing value; and (iv) a weighted estimating equation (WEE) model was used [32–34]. The WEE involved two steps. In the first step of the analysis, a sample selection model was constructed using baseline data to predict participation at each wave. On the basis of these models, probabilities of study participation were estimated using the MVPRED procedure of STATA. In the second step, data were re-analysed using a logistic GEE model on the full data with observations for each individual weighted by the inverse of the probability of study participation to adjust for sample selection bias. The results referring to assumptions (i) and (iii) about missing data are not reported in this paper and will be provided by the corresponding author on request.

## RESULTS

### Baseline characteristics

The baseline characteristics of the sample are shown in Table 2. Because our randomization procedure might have been biased due to excluding participants re-attending the practice within the later study conditions, we tested our study groups with respect to differences in baseline characteristics. There were no significant differences.

**Table 2:** Distribution of potential confounders; values are numbers (percentage) unless stated otherwise.

	Assessment only	Tailored letters	Brief advice	Total n	P*
All subjects	609	488	402	1499	
Socio-demographic variables					
Gender					0.204
Male	309 (50.7)	242 (49.6)	223 (55.5)	774	
Female	300 (49.3)	246 (50.4)	179 (44.5)	725	
Mean (SD) age	34.8 (13.4)	33.8 (13.2)	33.1 (12.5)	1499	0.228
Educational level					0.403
< 10 years of schooling	209 (34.3)	151 (30.9)	117 (29.1)	477	
= 10 years of schooling	294 (48.3)	256 (52.5)	217 (54.0)	767	
> 10 years of schooling	84 (13.8)	68 (13.9)	59 (14.7)	211	
No information	22 (3.6)	13 (2.7)	9 (2.2)	44	
Unemployed					0.396
Yes	86 (14.1)	62 (12.7)	66 (16.4)	214	
No	416 (68.3)	334 (68.4)	267 (66.4)	1017	
No information	107 (17.6)	92 (18.9)	69 (17.2)	268	
Married or living in stable partnership					0.717
Yes	409 (67.2)	323 (66.2)	279 (69.4)	1011	
No	186 (30.5)	152 (31.1)	118 (29.4)	456	
No information	14 (2.3)	13 (2.7)	5 (1.2)	32	
Health-related variables					
Mean (SD) self-rated health index EQ-VAS	70.7 (20.5)	69.9 (19.1)	69.7 (19.8)	1260	0.800
No information	103 (16.9)	74 (15.2)	62 (15.4)	239	
Reason for consulting the practitioner					0.764
Acute problems	283 (46.5)	225 (46.1)	195 (48.5)	703	
Chronic problems	140 (23.0)	115 (23.6)	97 (24.1)	352	
Preventive check-up	142 (23.3)	120 (24.6)	83 (20.6)	345	
No information	44 (7.2)	28 (5.7)	27 (6.7)	99	
Smoking-related variables					
Mean (SD) number of cigarettes smoked per day	16.2 (7.6)	16.4 (7.7)	16.9 (7.9)	1490	0.464
No information	4 (0.7)	4 (0.8)	1 (0.2)	9	
Mean (SD) FTND	3.3 (2.1)	3.1 (2.0)	3.3 (2.1)	1452	0.463
No information	26 (4.3)	15 (3.1)	6 (1.5)	47	
Intention to quit smoking					0.248
No intention	412 (67.7)	312 (63.9)	247 (61.4)	971	
Within next 6 months	160 (26.3)	133 (27.3)	128 (31.8)	421	
Within next 4 weeks	27 (4.4)	29 (5.9)	26 (6.5)	82	
No information	10 (1.6)	14 (2.9)	1 (0.2)	25	
24-hour quit attempt in the past year					
No attempt	426 (70.0)	340 (69.7)	274 (68.2)	1040	0.847
≥ 1 attempt	183 (30.0)	146 (29.9)	128 (31.8)	457	
No information	0 (0.0)	2 (0.4)	0 (0.0)	2	
Mean (SD) carbon monoxide in exhaled air p.p.m.	16.7 (10.2)	16.3 (9.4)	15.7 (9.5)	1350	0.506
No information	67 (11.0)	46 (9.4)	36 (9.0)	149	
Smoking partner					0.620
Yes	253 (41.5)	201 (41.2)	181 (45.0)	635	
No	138 (22.7)	113 (23.2)	86 (21.4)	337	
No partner	140 (23.0)	124 (25.4)	90 (22.4)	354	
No information	78 (12.8)	50 (10.2)	45 (11.2)	173	

* Bivariate comparison of valid cases adjusted for sampling design: Rao/Scott corrected χ^2^-statistic for categorical variables and adjusted Wald test statistic for continuous variables. FTND: Fagerstöm Test for Nicotine Dependence; SD: standard deviation; EQ-VAS: EuroQol visual analogue scale; p.p.m.: parts per million.

### Process evaluation

The preparation of the intended number of three tailored letters was possible only if sufficient data from the baseline, 3-month and 6-month assessments were available. Three hundred and six (62.7%) participants received three letters, 100 (20.5%) two, and 82 (16.8%) one. Self-help manuals were sent only if a tailored letter was available. At the 12-month follow-up 89.1% of the tailored letters intervention group confirmed receiving the letter or the self-help manuals. Among the assessment-only group, 3.5% falsely remembered receiving such material.

For each patient allocated to the brief advice group the practitioners rated the counselling activities on a form directly after the consultation. According to this, advice (i.e. a communication including active participation of the patient) was given to 353 (87.8%) participants. Additionally, smoking was at least addressed (i.e. the patient did not participate actively in the communication) in the consultation of 35 (8.7%) participants, leaving 14 (3.5%) participants without documented counselling activities of the practitioner. The practitioners estimated that the counselling lasted 7 minutes [standard deviation (SD) = 2.9] on average. Self-help manuals were handed out by the practitioner to 343 (85.3%) participants. At the 12-month follow-up 41.5% of the brief advice group remembered receipt of the self-help manuals.

Although training of the practitioners was held after the recruitment of the assessment-only and tailored letters group, these patients may have been counselled within later practice visits. Therefore, we compared previous counselling activities at baseline and 12-month follow-up. At baseline 24.0% of all participants reported that smoking had been addressed by their practitioner within the last 12 months. At the 12-month follow-up, 24.1% of the assessment-only group, 25.6% of the tailored letters group and 46.6% of the brief advice group reported that their practitioner had addressed smoking in the past year.

### Outcomes

Table 3 shows the point prevalence and prolonged abstinence for each follow-up. Prevalence figures were calculated by excluding all participants with missing follow-up data and by assuming that all participants with missing follow-up data were smokers, respectively. Abstinence increased by time in all study groups as well as the absolute difference of the abstinence rates between the intervention and the assessment-only groups. The highest rates were found in the tailored letters intervention group, followed by the brief advice intervention group and the assessment-only control group.

**Table 3:** Point prevalence and prolonged abstinence for different follow-ups across study groups.

		Assessment only			Tailored letters			Brief advice		
				
Outcome	Follow-up	n abstinent	% among those followed-up	% among those included at baseline	n abstinent	% among those followed-up	% among those included at baseline	n abstinent	% among those followed-up	% among those included at baseline
24-hour	6-month	28	5.8	4.6	39	11.8	8.0	24	7.5	6.0
abstinence	12-month	43	9.5	7.1	54	17.0	11.1	47	15.3	11.7
	18-month	59	14.2	9.7	77	25.6	15.8	53	18.9	13.2
	24-month	65	16.4	10.7	82	29.7	16.8	62	23.3	15.4
7-day	6-month	17	3.6	2.8	31	9.4	6.4	16	5.0	4.0
abstinence	12-month	38	8.4	6.2	44	14.0	9.0	37	12.1	9.2
	18-month	51	12.3	8.4	67	22.4	13.7	50	17.8	12.4
	24-month	57	14.4	9.4	75	27.2	15.4	57	21.4	14.2
4-week	6-month	15	3.1	2.5	22	6.7	4.5	13	4.0	3.2
abstinence	12-month	36	8.0	5.9	40	12.7	8.2	39	12.7	9.7
	18-month	48	11.6	7.9	63	21.1	12.9	45	15.9	11.2
	24-month	57	14.4	9.4	72	26.0	14.8	56	21.1	13.9
6-month	12-month	7	1.6	1.1	19	6.1	3.9	15	4.9	3.7
abstinence	18-month	25	6.0	4.1	36	12.0	7.4	34	12.1	8.5
	24-month	41	10.5	6.7	50	18.3	10.2	39	14.8	9.7

To explore the long-term stability of early intervention effects we calculated the percentage of participants reporting abstinence for the preceding 6 months at the 12-month and all subsequent follow-ups. When excluding participants missed at any of the three follow-ups, which were necessary to construct this measure, 1.5% of the assessment-only control group (five of 341), 6.0% (14 of 232) of the tailored letters intervention group and 3.6% (10 of 224) of the brief advice intervention group stopped smoking within the first 6 months and maintained abstinence until month 24. Assuming those participants missed at a follow-up to be smokers, the respective figures were 0.8% (five of 609), 2.9% (14 of 488) and 2.5% (10 of 402). For both assumptions about missingness the differences between the control and each of the intervention groups were statistically significant.

The GEE analyses allowed to test for intervention effects simultaneously across assessment times considering all available longitudinal information, i.e. each observation irrespective of non-participation at a previous or later follow-up time. We tested the robustness of our findings with respect to potential bias caused by missing follow-up observations. Table 4 shows the results from two of these approaches, i.e. a WEE analysis adjusting the outcome for different probabilities of participation in the follow-ups and a GEE analysis assuming those missed for follow-up to be smokers. All comparisons of the intervention groups with the assessment-only group were statistically significant. However, effect estimates for the tailored letters intervention are lower, particularly for the more rigid assumptions about missing data. Based on estimates from the crude analyses using the WEE approach, the odds of 7-day point prevalence and 6-month prolonged abstinence were 2.1 times higher in the tailored letters intervention group compared to the assessment-only control group. Comparison of the brief advice intervention group with the assessment-only control group revealed odds ratios of 1.6 and 1.9, respectively. The time variable was highly significant in all models, reflecting the increase of abstinence by time across all study groups. Analyses adjusting for the baseline characteristics of the participants showed minor changes (cf. right column of Table 3).

**Table 4:** GEE* analyses of treatment effects

			Crude analyses†		Adjusted analyses‡	
Procedure to take into account missing data	Outcome measure	Contrast (second group is reference)	P	OR (95% CI)	P	OR (95% CI)
Participants lost to follow-up assumed to be smokers	7-day point abstinence	Tailored letters versus assessment only	**0.001**	1.7 (1.3–2.4)	**0.002**	1.7 (1.2–2.4)
		Brief advice versus assessment only	**0.016**	1.5 (1.1–2.2)	**0.026**	1.5 (1.1–2.2)
		Tailored letters versus brief advice	0.470	1.1 (0.8–1.6)	0.522	1.1 (0.8–1.6)
	6-month prolonged abstinence	Tailored letters versus assessment only	**0.004**	1.9 (1.2–2.8)	**0.013**	1.7 (1.1–2.7)
		Brief advice versus assessment only	**0.005**	1.9 (1.2–2.9)	**0.011**	1.8 (1.1–2.9)
		Tailored letters versus brief advice	0.931	1.0 (0.6–1.5)	0.869	1.0 (0.6–1.5)
Weighted estimation equation adjusting for probability of follow-up participation	7-day point abstinence	Tailored letters versus assessment only	**< 0.001**	2.1 (1.5–3.0)	**< 0.001**	2.0 (1.4–2.9)
		Brief advice versus assessment only	**0.010**	1.6 (1.1–2.3)	**0.022**	1.6 (1.1–2.3)
		Tailored letters versus brief advice	0.158	1.3 (0.9–1.8)	0.177	1.3 (0.9–1.9)
	6-month prolonged abstinence	Tailored letters versus assessment only	**0.001**	2.1 (1.3–3.2)	**0.005**	1.9 (1.2–3.1)
		Brief advice versus assessment only	**0.004**	1.9 (1.2–3.1)	**0.009**	1.9 (1.2–3.0)
		Tailored letters versus brief advice	0.793	1.1 (0.7–1.6)	0.896	1.0 (0.7–1.6)

A complete table, including analyses of all outcome measures and all assumptions about missing data, is available on request.

* Generalized estimation equation models to predict smoking cessation taking into account cluster correlation within practices and within subjects across measurement periods. Models are based on an unstructured correlation structure for repeated observation within subjects. Six-, 12-, 18- and 24-month assessment have been considered for 7-day point abstinence and 12-, 18- and 24-month assessment for 6-month prolonged abstinence.

† Including the predictor variables study group and time.

‡ Including the predictor variables study group, time, age, gender, educational level, reason for consulting the practitioner, Fagerstöm Test for Nicotine Dependence, 24-hour quit attempt in the past year and intention to quit. CI: confidence interval; OR: odds ratio.

To test for a linear trend of the relative effect of the interventions across the different follow-up times we expanded our GEE main effect models by including time × intervention interaction terms. None of these analyses revealed a significant interaction.

Comparisons of both interventions revealed an odds ratio of 1.4 for 24-hour point prevalence abstinence for the GEE analysis, excluding participants lost to follow-up, indicating larger effects of the tailored letters [data not reported in Table 3: crude analysis *P* = 0.047, 95% confidence interval (CI) 1.0–1.9; adjusted analysis *P* = 0.037, 95% CI 1.0–2.0]. Differences approached significance for 7-day point prevalence abstinence [crude analysis *P* = 0.068, odds ratio (OR) = 1.4 95% CI 1.0–1.9; adjusted analysis *P* = 0.063, OR = 1.4, 95% CI 1.0–2.0]. With regard to prolonged abstinence, numerical differences were even lower and vanished completely for some of the analyses, taking into account the alternative assumptions about lost for follow-up. None were statistically significant.

## DISCUSSION

First, this study demonstrated that both interventions, i.e. up to three computer-tailored letters or a one-session practitioner-delivered brief advice, are effective in reducing the proportion of current smoking compared to no intervention. Secondly, the computer-tailored letters are at least equivalent to brief advice and tend to be even more effective with respect to point prevalence abstinence. Thirdly, by using a representative sample of practitioners and patients it seems appropriate to generalize findings to the entire population of patients and general practices.

With regard to the efficacy of the tailored-letter intervention in various populations, our study replicated results of studies using comparable recruitment procedures and follow-up periods [22,35,36]. As found previously in these studies, the cessation rates in our control group are beyond the rates that would have been expected for self-change based on population data. One possible reason might be that participation in a study focusing on smoking and recurrent assessments on psychological constructs related to smoking behaviour change might itself form a minimal-intervention [36]. Accordingly, a general practice-based smoking intervention study revealed that abstinence rates after 1 year were increased by 40% in an assessment-only compared to a no-assessment control group [37]. This effect might be particularly relevant for Germany, with currently low tobacco control measures and low counselling activities in primary medical care [38,39]. The relative intervention effects could therefore be underestimated by our results. In line with previous findings from German studies [40,41], the proportion of smokers not intending to quit is substantially higher compared to the proportion found in American studies. This could have been expected to be associated with lower quit rates and thus reduced intervention effects. On the other hand, our intervention may have capitalized on the patient–practitioner relationship and the favourable psychological state which has been discussed to be associated with the practice visit [42]. Thus, our results imply that implementing this class of interventions in primary care can be beneficial even in permissive smoking cultures, with a high proportion of smokers not intending to quit.

In contrast to our study, general practice-based studies on computer-tailored interventions in the United Kingdom have revealed less favourable results. By recruiting patients via mail the proportion of smokers who received treatment was considerable lower (42% [20]; 9% [21]) compared to our study using personal recruitment within practices. This selection process of the previous studies may limit the generalizability for the entire population of smoking patients. Differences in study design might also explain lower intervention effects: (i) both previous studies relied on biochemically confirmed abstinence. We refrained from this approach because these studies demonstrated that collecting saliva samples is not feasible, even though validation had been restricted to quitters (rate of analysed saliva samples among claimed quitters in previous research: 63% [20]; 31% [21]). Given the low intensity of the intervention and low demand characteristic in our study, the validity of self-reported smoking behaviour is more comparable to epidemiological than clinical studies with stronger interpersonal involvement, and thus denial of smoking is less likely [43]; (ii) one of the UK studies was restricted to one tailored letter, which precludes feedback on intra-individual change over time and provision of behavioural strategies if smokers not ready to quit move to action [20]. The other study provided a maximum of three letters, but the number of patients receiving the full intervention dose was substantially lower than in our study [21]. Although one study testing the efficacy of different numbers of intervention letters did not find a clear dose–response relationship [44], a meta-analysis of physician-delivered advice revealed that studies including follow-up interventions are more effective [2].

A strength of the brief advice intervention is that it is highly feasible [14]. Individual training sessions held at each practice site allowed us to implement the intervention in almost all randomly selected practices. For the period of 1 week and with the support provided by our research nurse, the vast majority of eligible patients received counselling within regular practice hours. The relative effect of the brief advice compared to the assessment-only condition was close to results reported by a recent meta-analysis pooling studies about physician advice for smoking cessation [2]. Due to the high overall quit rate in our study, the absolute difference in cessation is above the 2.5% found in this meta-analysis. We tried to standardize and structure the brief advice by means of training and materials (i.e. information sheet on patients smoking-related characteristic, desktop resource). However, our study design did not allow us to evaluate the particular activities delivered by the practitioners. More active learning methods, e.g. role-plays or context-bound training, are likely to be more effective [45]. On the other hand, a higher intensity of training can be expected to decrease the participation rate of practitioners.

Taking population impact as a yardstick, several aspects have to be considered when comparing the interventions included in our study. The efficacy of the tailored-letters intervention tends to be superior for all outcome measures. However, statistical significance was reached for only one outcome measure and was not robust for different assumptions regarding missing data. Interpretation of these findings is complicated by the fact that there is a considerable overlap of the study conditions. Both interventions included the same self-help manuals and perhaps training improved the quality or quantity of advice for all patients, although the data on frequency of advice giving in the year prior to the intervention and following it suggest no effect on quantity. Thus, the assessment-only and tailored-letters group may also receive advice exceeding routine care. When testing minimal interventions for all smokers, even small differences are important and thus future studies including larger samples are needed to establish differences between both interventions. With respect to broad implementation, cost-effectiveness needs further consideration in future research. With appropriate technology, the tailored-letters intervention might use fewer resources than brief advice, which involves high costs for the working time of the practitioner.

Further limitations of our study are: (i) in Germany, nicotine replacement medication is not reimbursed by health insurance companies. Although our interventions included the recommendation of nicotine replacement therapy for smokers intending to quit, only a small proportion of participants (5.7%) reported use of such medications at any follow-up. Providing free nicotine replacement medication might increase the efficiency of our interventions [46,47]. (ii) The practices included in our study received a fair amount of support (i.e. presence of a study nurse in each practice, organizing the screening and baseline assessment; administration of the tailored-letter intervention by our research staff) and systematic implementation of each intervention was limited to 1 week. Therefore, integrating interventions into routine care and broad implementation may need to consider further structural and organizational factors (e.g. adequate reimbursement of the practitioner, distribution of intervention materials and training for practitioners and their staff, implementation and maintenance of adequate computer hard and software). (iii) A considerable proportion of participants were lost to follow-up and these proportions vary across study groups. This is mitigated by the fact that about three-quarters of the patients not completing the follow-up were lost because we were unable to contact them and only less than 10% refused. Further several procedures to account for possible non-response bias did not change our findings. (iv) We utilized a quasi-randomization procedure based on time of practice attendance and fixed sequence of study groups. Thus, patients attending the practice frequently are under-represented in the later study groups. However, we found no significant baseline differences, and adjusting our analyses for baseline characteristics revealed the same pattern of results. (v) Due to seasonal effects, the numbers of patients registered were lower in the intervention conditions. Thus the work-load of the practitioners, particularly when providing the brief advice, was slightly lower than average.

In conclusion, both of the two minimal interventions have the potential to substantially reduce smoking in entire populations of smoking patients. The long-term intervention effects observed in our study demonstrated that the clinician's efforts to address smoking cessation are a reasonable investment of time, even if immediate effects are not obvious. Interventions based on computer expert system technology can become a valuable extension to established treatment opportunities. In the light of limited resources and increasing demands with respect to preventive measures assigned to general practitioners [48], computer-generated tailored letters are a promising option to overcome the existing barriers to routinely provide smoking cessation interventions.
